# Spontaneous emergence of chirality in achiral lyotropic chromonic liquid crystals confined to cylinders

**DOI:** 10.1038/ncomms9067

**Published:** 2015-08-19

**Authors:** Karthik Nayani, Rui Chang, Jinxin Fu, Perry W. Ellis, Alberto Fernandez-Nieves, Jung Ok Park, Mohan Srinivasarao

**Affiliations:** 1 School of Materials Science and Engineering, Georgia Institute of Technology, Atlanta, Georgia 30332, USA; 2 School of Chemical and Biomolecular Engineering, Georgia Institute of Technology, Atlanta, Georgia 30332, USA; 3 School of Physics, Georgia Institute of Technology, Atlanta, Georgia 30332, USA; 4 School of Chemistry and Biochemistry, Georgia Institute of Technology, Atlanta, Georgia 30332, USA; 5 Center for Advanced Research in Optical Microscopy, Georgia Institute of Technology, Atlanta, Georgia 30332, USA

## Abstract

The presumed ground state of a nematic fluid confined in a cylindrical geometry with planar anchoring corresponds to that of an axial configuration, wherein the director, free of deformations, is along the long axis of the cylinder. However, upon confinement of lyotropic chromonic liquid crystals in cylindrical geometries, here we uncover a surprising ground state corresponding to a doubly twisted director configuration. The stability of this ground state, which involves significant director deformations, can be rationalized by the saddle-splay contribution to the free energy. We show that sufficient anisotropy in the elastic constants drives the transition from a deformation-free ground state to a doubly twisted structure, and results in spontaneous symmetry breaking with equal probability for either handedness. Enabled by the twist angle measurements of the spontaneous twist, we determine the saddle-splay elastic constant for chromonic liquid crystals for the first time.

## Abstract (web summary)

Chirality in molecular materials is commonly used to manipulate the polarization of light. Here, Nayani *et al*. observe the formation of doubly twisted structure in achiral chromonic liquid crystals when confined to a cylindrical capillary, which leads to spontaneous chiral breaking.

## Graphical abstract

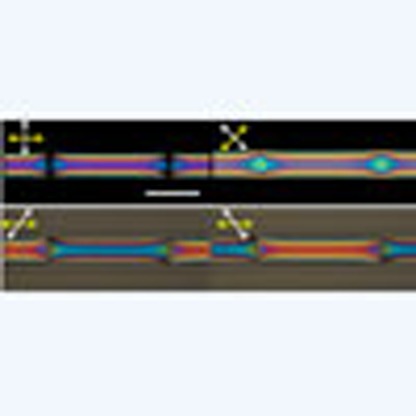

Pasteur's brilliant experiments dealing with the crystallization of tartrates provided the first glimpse of spontaneous mirror-symmetry breaking that led to the foundation of stereochemistry^1^. Optical activity, a consequence of reflection-symmetry breaking, since the time of its discovery by Biot in the early 1800s, has captivated the imagination of scientists^2^,^3^,^4^. Since Pasteur's time, the appearance of macroscopic chirality during crystallization from both chiral and achiral molecules has been frequently observed^5^. Crystallization apart, optical activity resulting from achiral units has been observed in liquid crystals and polymeric materials without a molecular chiral centre. Chirality, indeed, has been an important feature of liquid crystals since the time of their discovery in 1888 (refs 6, 7).In particular, the appearance of chirality from achiral molecules has been the subject of study for the past two decades stimulated by the synthesis of bent-core molecules that were found to exhibit chiral domains in their fluid state^8^. Confinement can also result in spontaneous reflection-symmetry breaking; this has been seen in droplets^9^,^10^,^11^,^12^, tactoids^13^,^14^ and in toroids^15^.

Here we explore an unexpected spontaneous reflection-symmetry breaking when a class of liquid crystals, known as lyotropic chromonic liquid crystals (LCLCs), are confined to cylindrical capillaries. In describing the kind of mirror-symmetry breaking that we are reporting on has historically been referred to as ‘chiral symmetry breaking'. It should, however, be noted that it is inappropriate to use this term when describing the emergence of chirality from achiral systems, as has been pointed out almost a decade ago^16^,^17^. Hence, we use the term ‘reflection-symmetry breaking' in keeping with the suggestion made by Barron^18^.

When nematic liquid crystals are confined within flat boundaries with planar boundary conditions, the ground state is characterized by an average molecular orientation along a specific direction referred to as the easy axis of the director. Confinement of nematics to curved geometry results in a much richer phenomenology^19^. Curvature for nematics costs energy. This can be appreciated when one notices that the elastic free-energy expression of the director **n** is of the order of (∇**n**)^2^ (ref. 20). For this reason, the Frank free energy is oftentimes referred to as the curvature elasticity of nematic liquid crystals^21^. This richness is further enhanced if the nematic has significant anisotropy of its elastic constants^10^,^13^,^22^. In this study, we show the emergence of spontaneous chirality when achiral chromonic molecules are confined to a cylindrical geometry. The intricate nature of the coupling of curvature to the free energy and sufficient anisotropy of elastic constants drive the observed spontaneous reflection-symmetry breaking transition.

LCLCs are composed of plank-like molecules with a poly-aromatic core and polar peripheral groups. The poly-aromatic cores stack face to face governed by *π*–*π* interactions^23^,^24^. At low concentrations, the aggregates are short and orient randomly. As the concentration increases, the aggregates increase in length and eventually form a nematic phase. Although Onsager's excluded volume theory would be the intuitive way to describe the isotropic–nematic transition of these lyotropic systems, it has been found that the critical concentration of the isotropic–nematic transition is much lower than that predicted by Onsager. Several models such as Y-stacks and slip stacks have been suggested to address the discrepancy^25^,^26^. Another curious aspect of these phases is the fact that the twist elastic constant (*K*_22_) is an order of magnitude lower than the splay (*K*_11_) and bend (*K*_33_) constants^27^. In this regard, LCLCs bear striking similarity with several other polymeric nematics whose *K*_22_/*K*_11_ and *K*_22_/*K*_33_ are of the same order of magnitude as LCLC's^28^,^29^,^30^,^31^. This seems to be a feature common to nematics whose constitutent nematogens are composed of semiflexible units. However, we are unaware of any first principles study in the literature that addresses the reason for the twist elastic constant in these systems being curiously low. These fascinating features of LCLCs distinguish them from small-molecule thermotropic nematics, thus conferring on confined LCLCs new and richer phenomenology.

Spontaneous reflection-symmetry breaking has been observed previously in tactoids of disodium cromoglycate (DSCG)^32^. Competition of the twist and splay deformation energies has been used to describe the emergence of chirality in the twisted tactoids. Such an argument is particularly valid in the case of LCLCs due to the twist elastic constant being an order of magnitude smaller than the splay elastic constant. Similarly spherical droplets of Sunset Yellow FCF (SSY) with planar boundary conditions also break reflection symmetry by relieving the costly splay close to the surface defects (boojums) with a twist deformation^33^. In both of these cases, any permitted ground state with or without twist involves a deformation of the director. The uniqueness of our work lies in the fact that when confined to a cylindrical geometry with planar anchoring, we uncover a configuration with significant distortions, even though there exists a configuration free of director deformations, the axial configuration, where the director lies along a cylindrical axis^34^. This is a truly remarkable observation, which imparts novelty to the current work. We demonstrate that the often-neglected saddle-splay (*K*_24_) contribution is crucial in stabilizing the observed twisted director profile in cylindrical capillaries. Provided there exists sufficient anisotropy between the saddle-splay and the twist elastic constants, the saddle-splay term not only screens the twist but also stabilizes the director deformation by decreasing the free energy of the twisted configuration below that of the deformation-free structure^35^.

## Results

### Polarized optical microscopy

**Figure 1:**
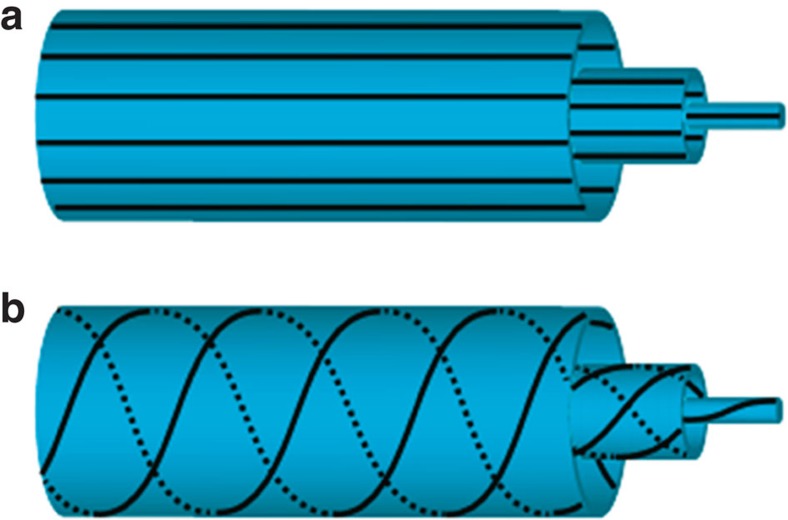
Schematics of director configurations in a cylindrical capillary. (**a**) Axial director profile. (**b**) Doubly twisted director profile.

**Figure 2:**
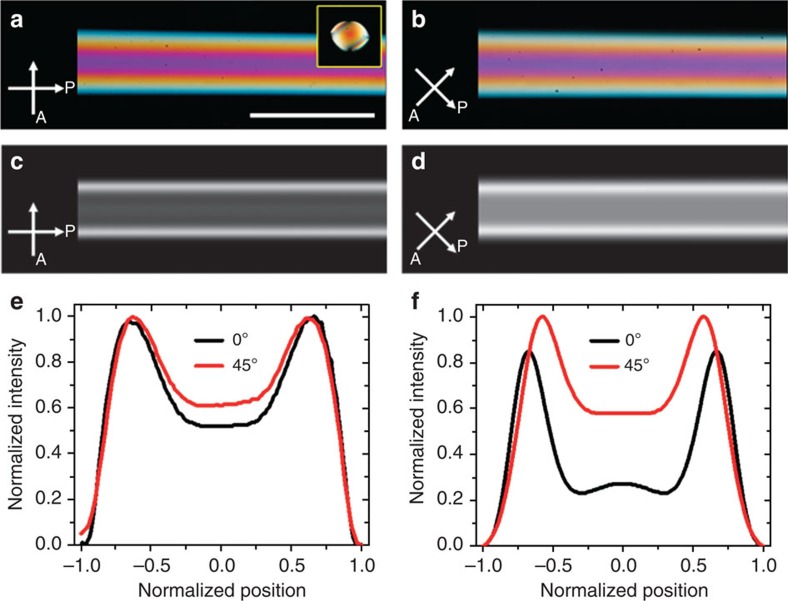
Experimental and simulated images of doubly twisted director in a cylinder. (**a**,**b**) POM images of DSCG in a cylindrical capillary. Scale bar, 100 μm. The inset shows a bipolar tactoid of DSCG nucleating on the capillary wall in the biphasic region. (**c**,**d**) Simulation images of double twist configuration. (**e**) Intensity profiles of the cross-sections of **a** (black line) and **b** (red line). (**f**) Intensity profile of the cross-section of **c** (black line) and **d** (red line).

Consider an axial director profile as illustrated in Fig. 1a. For this director configuration the intensity profile of the transmitted light in a crossed polarized setup is of the form: 
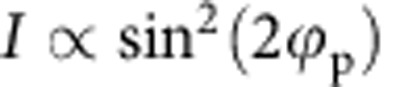
, where 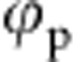 is the angle between the director and the polarizer^36^. The intensity is a minimum when the director is either parallel or perpendicular to the incident polarization, corresponding to complete extinction of the light. The maxima in the intensity correspond to the cases where the director makes an angle of 45° with respect to the incident polarization direction. Polarized optical microscopy (POM) images of two angular positions of a capillary filled with DSCG are shown in Fig. 2a,b, which correspond to the long axis of the capillary being parallel and at 45° with respect to the incident polarization direction, respectively. It should be noted that neither one of these situations display the expected extinction of the incident light. This remarkable feature points to the fact that we must have a twisted structure in place of an axial one. In an axial configuration, the image corresponding to Fig. 2a would be one of complete extinction of light. Instead, we see that the intensity in both Fig. 2a and b are comparable. This implies that the plane of polarization of the incident light is rotated by the liquid crystal, and upon exiting the capillary it is at an angle to the analyser that is not 0° or 90°. This is a classic signature of twist. A doubly twisted configuration, which could explain the observed experimental images is illustrated in Fig. 1b. The director of this configuration is axial at the centre of the cylinder and twists progressively as it approaches the surface. We confirm that the anchoring at the surface of the cylinder is planar by studying the tactoids that nucleate when the LCLC is in the biphasic region. The inset of Fig. 2a shows a bipolar tactoid, which serves as a confirmation that the surface anchoring is planar.

### Jones matrix simulations

To test the hypothesis of double twist, we perform Jones matrix simulations with a simple doubly twisted director ansatz. Jones matrices quantify the change in the polarization state of the light as it traverses the sample^36^,^37^. The director field is specified by *n=n*_*r*_*e*_*r*_*+n*_*θ*_*e*_*θ*_*+n*_*z*_*e*_*z*_ where *e*_*r*_*, e*_*θ*_ and *e*_*z*_ are the orthonormal vectors in cylindrical coordinates, with *n*_*r*_=0, 
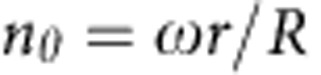
, 
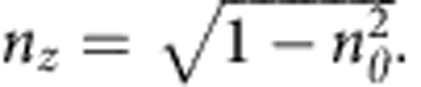
The twist parameter *ω* determines the amount of twist in the system; *ω=0* corresponds to an axial configuration. *r* is the radial distance from the centre of the circular cross-section and *R* is its radius. Indeed, the simulated POM textures conform to the experimental observations that the light is not extinguished when the capillary long axis is parallel to the polarization of the incident light, which is evident from Fig. 2c. Further, we note that the intensity when the angle between the incident polarization direction and the cylindrical long axis is 45°, *I*_45_, is always greater than the intensity when the angle between the incident polarization and the cylindrical long axis is 0°, *I*_0_, which is also in agreement with the experimental observations. Furthermore, the intensity profiles along the cross-section of the sample of both experiments and simulations are consistent with each other, as shown in Fig. 2e,f. The simulations capture the important characteristics of the experimental profiles. The consistency of simulation and experiments indicates that the doubly twisted configuration likely reflects molecular arrangement inside the capillary.

Having shown the persistence of a twisted structure with both experiments and simulations, we now address several consequent questions: what is the driving force for the spontaneous twist deformation of the director field? What is the magnitude of the twist? Does the magnitude of twist depend on the size of the capillary?

### Role of Saddle Splay Elasticity

We address the question of the driving force for the doubly twisted configuration over the axial configuration by considering the contribution of the saddle-splay (*K*_24_) term in the free-energy expression:


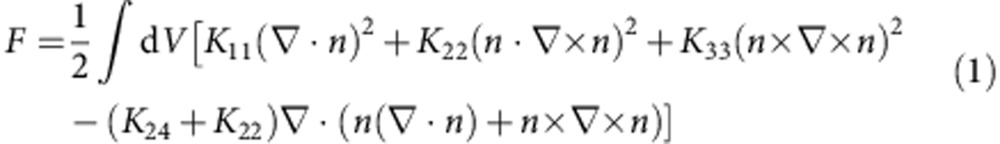


where *K*_11_, *K*_22_ and *K*_33_ are the Frank elastic constants associated with splay, twist and bend bulk deformations, respectively. The splay-bend (*K*_13_) contribution has been a contentious topic in nematic elasticity, but we can safely neglect the *K*_13_ term in our study as we are dealing with the case of planar anchoring. The role of the saddle-splay term, which is weighted with the elastic constant *K*_24_, is subject to great debate^38^. Several studies have neglected the contribution of saddle splay under strong anchoring conditions. This argument stems from the fact that the bulk integral of the saddle splay can be reduced to a surface integral using Stoke's theorem, and that the contribution of saddle splay would be negligible as the divergence contributions are small when we approach the infinitely removed surface. However, this argument is rather misguided as the saddle-splay contribution can only be neglected if the director depends only on one Cartesian coordinate^39^,^40^. For planar anchoring, this implies that when the confining boundaries are curved, the contribution of *K*_24_ cannot be trivially neglected. Further, in geometries such as cylinders where the two principal curvatures are very different, the contribution of saddle splay can play a pivotal role in determining the director configuration.

We highlight the crucial role played by the saddle-splay term in the spontaneous reflection-symmetry breaking of the director configuration resulting in a doubly twisted configuration for cylindrical confinement. For the case of planar anchoring, the saddle-splay term tends to align the director along the direction of largest principal curvature. This can be better understood when the contribution of *K*_24_ to the free energy per unit length is written in the form^41^: 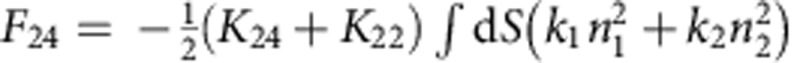, where *k*_1_ and *k*_2_ are the principal curvatures at a point on the surface, and *n*_1_ and *n*_2_ are the director components along the corresponding directions (see Supplementary Information). For a cylinder, *n*_1_=*n*_*θ*_ and *n*_2_=*n*_z_; hence *k*_1_=1/*R* and *k*_2_=0. As a result, for the case of cylindrical geometry, the integral of *F*_24_ is minimized when the director at the surface is along the *e*_*θ*_ direction. This drives the system to twist and provided that there is sufficient anisotropy of the elastic constants, the twisted structure is always stable. We tend to this argument with a simple theoretical model below.

The same ansatz, which was used for the Jones matrix simulations, is used for the theoretical calculations. We obtain an expression for the free energy per unit length as:


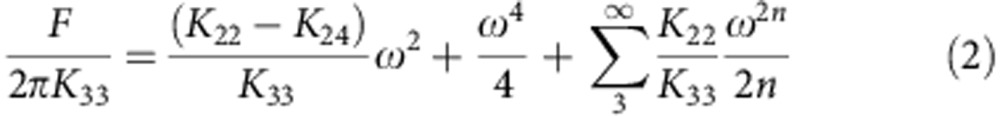


The form of the free energy is completely analogous to the Landau theory of magnetism^20^. It is, in fact, the sign of the quadratic term which determines whether the system adopts an axial configuration or breaks reflection symmetry, resulting in a doubly twisted director configuration. This theory is generic and would describe the reflection-symmetry breaking of any nematic fluid. Provided *K*_24_>*K*_22_, the director is always going to be twisted. For LCLCs, the low value of *K*_22_ confer them with the possibility of satisfying the twisting criterion. We can conclude that the value of *K*_24_ for the LCLCs used in the experiments is always greater than *K*_22_, as the director is twisted in our experiments. Although our model is simplistic in terms of the ansatz used, we capture the essential physics with the criterion that *K*_24_>*K*_22_ for a twisted structure.

### Waveguiding experiments

**Figure 3:**
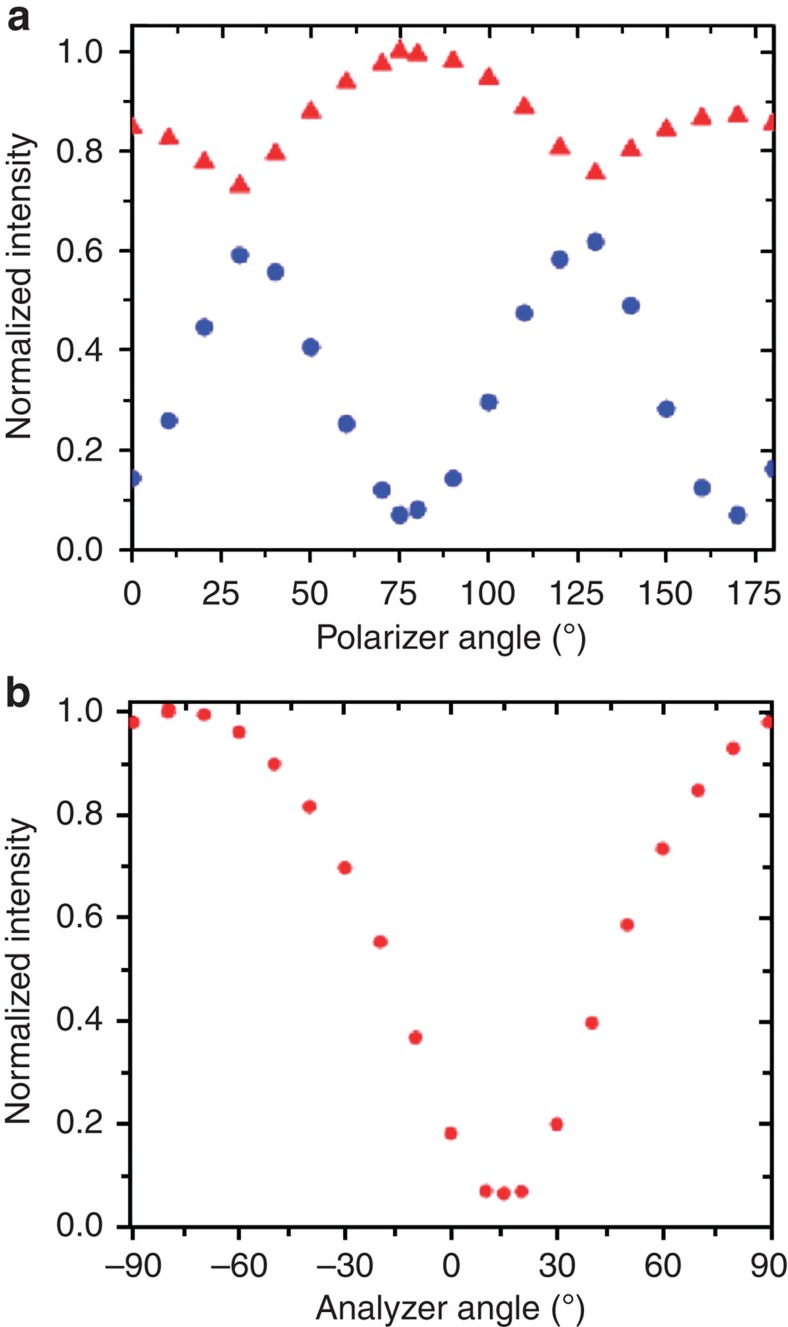
Intensity profiles from waveguiding measurements. (**a**) The maximum (red triangle dots) and minimum (blue circles) transmitted intensities at every polarizer angle when the analyser is rotated by 180°. (**b**) The transmitted intensity at every analyser angle when the polarizer is fixed at 75°.

To confirm the twisting criterion, we obtain the value of *K*_24_ by inserting the value of the twist parameter *ω* measured experimentally in the expression obtained by minimizing equation 2. We determine the twist parameter by measuring the twist angle as one traverses the diameter of the cylinder. In the Mauguin limit, where 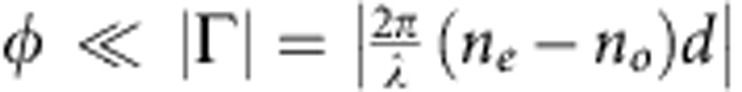, with *φ* the total twist angle and Γ the retardation caused by the anisotropy of the refractive indices^42^, the director field serves as a waveguide to the incident polarized light. As the birefringence, *n*_*e*_−*n*_*o*_, is fairly low (∼0.02) for DSCG, we use capillaries of diameter 400 μm to satisfy the waveguiding requirement. When the waveguiding criterion is met, the minimum in transmitted intensity corresponds to the situation where, the entry polarization of the incident light is along the long axis (extraordinary waveguiding) or short axis (ordinary waveguiding), and the analyser is perpendicular to the exit direction of the light. Theoretically, this corresponds to zero transmitted intensity. To determine this specific polarizer analyser combination, we first rotate the analyser every 5° at a given polarizer orientation. Then, the polarizer is rotated by 5° and the analyser rotation is repeated for the new polarizer orientation. The red triangles and blue circles in Fig. 3a show the maximum and minimum intensities, respectively, so obtained for the different polarizer orientations. It is evident from Fig. 3a, the minimum transmitted intensity corresponds to a polarizer angle of 75° for DSCG. For this polarizer orientation, the transmitted intensity as a function of the analyser rotation is shown in Fig. 3b. From the minimum in the plot of Fig. 3b, we estimate that the total twist angle is (150±10)°. Since, within our ansatz, *φ*=2 sin^−1^(*ω*), we find that *ω*=0.95±0.03. Substituting the value of *ω* thus determined into the theoretical expression below, which is obtained by minimizing the free-energy expression given by equation 2:


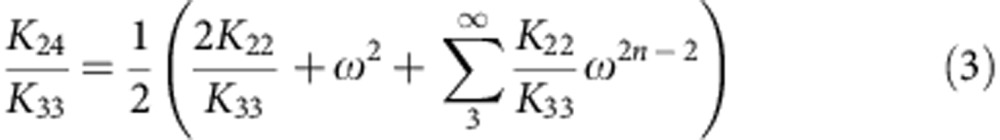


we find that the range of 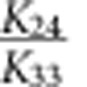 for DSCG is between 0.75 to 1.75 under the assumption that 
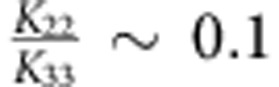
. We also perform the same experiment with SSY and obtain a twist angle close to 170°. This would correspond to a value of 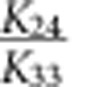 of 6.75. We note that the exact value of 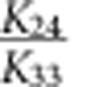 is extremely sensitive to the value of the twist angle for large twists (*ω*∼1). In comparison with small-molecule liquid crystals such as 5CB, we note that the value of *K*_24_ is significantly higher in comparison with the other elastic constants. For all the capillary dimensions (50, 100, 400 and 500 μm) used in the experiments, the twist angle measured is independent of the system size. This is another interesting aspect of our experimental system. Theoretically, we envision that very large values of twist per unit length can be obtained by cylindrical confinement of LCLCs. As the twist angle is independent of the system size, we can potentially confine LCLCs in as small a capillary as experimentally permitted and still obtain the same values of the twist angle. This makes our system a viable playground for a number of applications. We note that it is only the ratio of the elastic constants that dictates the magnitude of twist in the system. The obtained values of 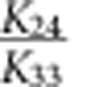 are at odds with Ericksen's inequalities^43^; these emerge on the basis that the Frank free energy is positive definite, with the minimum corresponding to a deformation-free director. In our experiments, the anisotropy of the elastic constants results in the formation of a ground state that is lower in energy than the deformation-free ground state. Hence, Ericksen's inequalities no longer apply.

**Figure 4:**
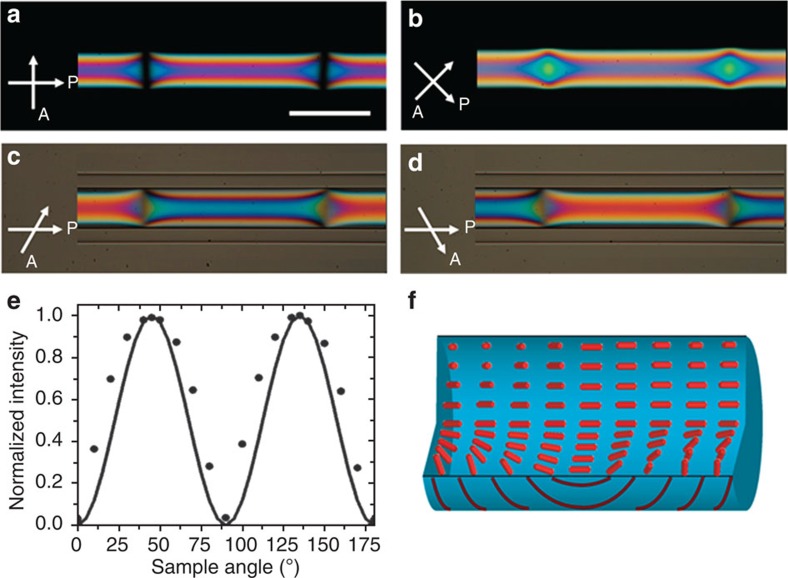
Neel walls separating oppositely handed domains. POM images of DSCG in cylindrical capillaries with Neel Walls separating domains of opposite twist when the polarizer and analyser make an angle of (**a**,**b**) 90° and (**c**,**d**) 60°. The opposite twist handedness is revealed from the complementary intensities at complementary polarizer- analyzer angles. White arrows represent the direction of polarizer (P) and analyser (A). Scale bar, 100 μm. (**e**) The intensity of the centre of the wall region as the sample is rotated under crossed polarizers (black circles) and the theoretical intensity profile of an axial configuration (black line). (**f**) Corresponding schematic of the director configuration in the wall region.

The sign of the twist parameter *ω* determines the handedness of the system as the free energy is invariant to sign inversion. This implies that there is equal probability of finding domains of either handedness. Indeed, we see that there are multiple domains in a single capillary. Furthermore, these domains have opposite handedness and are separated by Neel walls^44^. Figure 4a demonstrates walls separating oppositely handed domains, where the sample is along the polarizer in a crossed polarizer setup. We note the extinction of the transmitted light from the centre of the wall, indicating that it could be a Neel wall. In Fig. 4b, where the sample makes an angle of 45° with the polarizer, the transmitted intensity is a maximum from the wall region. The intensity profile of the centre of the wall as the sample is rotated under cross polarizers is reminiscent of an axial configuration as shown in Fig. 4e. This confirms that we, in fact, have a Neel Wall separating domains of opposite handedness, as illustrated in Fig. 4f. We establish that the regions separated by the Neel wall are oppositely twisted from complementary polarizer and analyser angles. For instance, the 60° and −60° as shown in Fig. 4c,d, the domains show exactly complementary intensity profiles, which is a signature of opposite handedness of twist.

## Discussion

We demonstrate the emergence of spontaneous chirality when LCLCs are confined to cylinders with degenerate planar anchoring conditions. Remarkably, the doubly twisted state is the chosen ground state even when there is a director configuration free of deformations. It is the contribution of the saddle-splay term drives the director to have a twisted structure. In addition our experiments present a unique and relatively straightforward path to measure the elastic constant *K*_24_ for LCLCs. This marks the first measurement of *K*_24_ for LCLCs. There is significant interest in the area of chiral separation of organic molecules in water. It has not escaped our attention that this configuration is an elegant platform for the detection and separation of chiral substances and enantiomers^45^. We have shown that the LCLC has equal probability of acquiring one handedness, and domains of opposite handedness are separated by a Neel wall. These experiments highlight the fascinating aspect of highly anisotropic elastic constants, a distinctive feature of LCLCs. This feature coupled with the curved geometry results in various fascinating phenomena and hints towards richer phenomenology when LCLCs are confined in more exotic geometries. Note that at the time of revision, we came across a similar report demonstrating reflection-symmetry breaking of LCLCs in cylindrical geometries^46^.

## Methods

### Experimental details

Aqueous solutions of SSY and DSCG were used in this study. Cylindrical capillaries (Vitrocom) of diameter 50, 100, 400 and 500 μm were used without any surface treatment. The capillaries were filled with 14, 16 and 18 wt% of DSCG and 30 wt% SSY by capillary force and then immediately sealed with epoxy to prevent water evaporation. The director profiles were interrogated using POM (Leica DMRX Microsystem).

### Atomic force microscopy measurements of the inner surface of the capillaries

**Figure 5:**
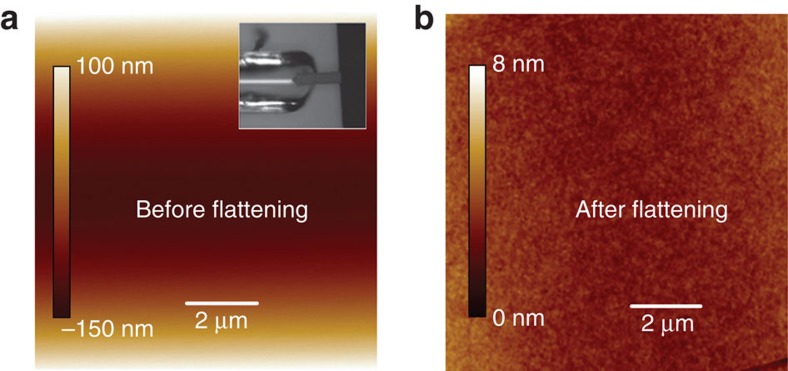
AFM measurements of the inner surface of the capillary: (**a**) Inner surface height profile of a 50-μm-diameter cylindrical capillary, the inset shows the AFM tip scanning the bottom of the inner wall of the capillary. (**b**) The surface tomography of the inner wall after flattening the image (**a**).

In order to rule out any surface imperfections that might result in twist, such as grooves inside the capillary, we performed AFM (NanoScope, BRUKER) measurements on the inner surface of the cylindrical capillary. Figure 5a is the surface height profile, from which the curvature of the capillary can be seen. We then flatten the image (Fig. 5a) using the software provided by the manufacturer to take away the global curvature and get the surface tomography as shown in Fig. 5b. There are no grooves or other structures on the inner wall that can provide a preferred surface anchoring for the liquid crystal.

## Additional information

**How to cite this article:** Nayani, K. *et al*. Spontaneous emergence of chirality in achiral lyotropic chromonic liquid crystals confined to cylinders. *Nat. Commun.* 6:8067 doi: 10.1038/ncomms9067 (2015).

## Supplementary Material

Supplementary Information Supplementary Note 1
