# Supplementary material for: Spontaneous emergence of chirality in achiral lyotropic chromonic liquid crystals confined to cylinders
Source: Nat Commun. 2015 Aug 19;6:8067. doi: 10.1038/ncomms9067 (PMC4560794; doi:10.1038/ncomms9067)
Supplement: Supplementary Information — Supplementary Note 1 [file ncomms9067-s1.pdf]

Supplementary Note 1

Derivation of the contribution of saddle- splay term to the free energy:

We start from the expression of Frank's free energy

$$F = \frac{1}{2}\int dV[K_{11}(\nabla \cdot \boldsymbol{n})^2 + K_{22}(\boldsymbol{n} \cdot \nabla \times \boldsymbol{n})^2 + K_{33}(\boldsymbol{n} \times \nabla \times \boldsymbol{n})^2 - (K_{24} + K_{22})\nabla \cdot (\boldsymbol{n}(\nabla . \boldsymbol{n}) + \boldsymbol{n} \times \nabla \times \boldsymbol{n})]$$

Contribution of the saddle- splay term is:

$$F_{24} = -\frac{1}{2}(K_{24} + K_{22}) \int dV[\, \nabla \cdot (\boldsymbol{n}(\nabla . \boldsymbol{n}) + \boldsymbol{n} \times \nabla \times \boldsymbol{n})]$$

Converting it to a surface integral using Stoke's theorem:

$$F_{24} = -\frac{1}{2}(K_{24} + K_{22}) \int dS[\, \nu \cdot (\boldsymbol{n}(\nabla . \boldsymbol{n}) + \boldsymbol{n} \times \nabla \times \boldsymbol{n})]$$

Where, ν is the unit normal vector of the surface. For the case of planar anchoring, $\nu . \boldsymbol{n}$ =0, so the first term drops out leaving us with:

$$F_{24} = -\frac{1}{2}(K_{24} + K_{22}) \int dS[\nu \cdot (\boldsymbol{n} \times \nabla \times \boldsymbol{n})]$$

Consider $\boldsymbol{n} \times \nabla \times \boldsymbol{n}$**:**

$(n \times \nabla \times n)_a = \epsilon_{abc}\, n_b\, \epsilon_{cpq}\, \partial_p n_q = (\delta_{ap}\delta_{bq} - \delta_{aq}\delta_{bp}) n_b \partial_p n_q = -n_b \partial_b n_a$

Also, from product rule

$\nu_b \partial_a n_b + n_b \partial_a \nu_b = \partial_a(\nu_b n_b) = 0$

So we can re-write the contribution of F_{24} as:

$F_{24} = -\frac{1}{2}(K_{24} + K_{22}) \int dS[\nu \cdot (n . \nabla) n]$

Defining the extrinsic curvature tensor as:

$L_{ij} = e_i . (e_j . \nabla)\nu$, where e_1 and e_2 are the directions of principal curvatures k_1 and k_2.

$F_{24} = -\frac{1}{2}(K_{24} + K_{22}) \int dS n_i L_{ij} n_j$;

From the definition of the principal curvature we have

$F_{24} = -\frac{1}{2}(K_{24} + K_{22}) \int dS(k_1 n_1^2 + k_2 n_2^2)$
